# The immune consequences of preterm birth

**DOI:** 10.3389/fnins.2013.00079

**Published:** 2013-05-21

**Authors:** Jacqueline M. Melville, Timothy J. M. Moss

**Affiliations:** ^1^The Ritchie Centre, Monash Institute of Medical Research, Monash UniversityClayton, VIC, Australia; ^2^Department of Obstetrics and Gynaecology, Monash UniversityClayton, VIC, Australia

**Keywords:** preterm, immature immune system, inflammation, antenatal glucocorticoids, ventilation

## Abstract

Preterm birth occurs in 11% of live births globally and accounts for 35% of all newborn deaths. Preterm newborns have immature immune systems, with reduced innate and adaptive immunity; their immune systems may be further compromised by various factors associated with preterm birth. The immune systems of preterm infants have a smaller pool of monocytes and neutrophils, impaired ability of these cells to kill pathogens, and lower production of cytokines which limits T cell activation and reduces the ability to fight bacteria and detect viruses in cells, compared to term infants. Intrauterine inflammation is a major contributor to preterm birth, and causes premature immune activation and cytokine production. This can induce immune tolerance leading to reduced newborn immune function. Intrauterine inflammation is associated with an increased risk of early-onset sepsis and likely has long-term adverse immune consequences. Requisite medical interventions further impact on immune development and function. Antenatal corticosteroid treatment to prevent newborn respiratory disease is routine but may be immunosuppressive, and has been associated with febrile responses, reductions in lymphocyte proliferation and cytokine production, and increased risk of infection. Invasive medical procedures result in an increased risk of late-onset sepsis. Respiratory support can cause chronic inflammatory lung disease associated with increased risk of long-term morbidity. Colonization of the infant by microorganisms at birth is a significant contributor to the establishment of the microbiome. Caesarean section affects infant colonization, potentially contributing to lifelong immune function and well-being. Several factors associated with preterm birth alter immune function. A better understanding of perinatal modification of the preterm immune system will allow for the refinement of care to minimize lifelong adverse immune consequences.

Preterm birth is defined as birth prior to 37 completed weeks of gestation (Beck et al., 2010); term is 40 weeks. Fifteen million neonates, 11% of the world's births, are born preterm every year (Howson et al., 2012). The incidence of preterm birth is as high as 15% in many countries (Howson et al., 2012). In Australia 8% of babies are born preterm (Laws et al., 2007; Li et al., 2011). Preterm birth accounts for 35% of all neonatal deaths worldwide: over one million every year (Howson et al., 2012).

Survivors of preterm birth face lifelong morbidity, with increased risks of cerebral palsy, intellectual disability (Doyle, 2001; Mwaniki et al., 2012), respiratory disease (Jobe et al., 2008) and vision impairment (O'Connor et al., 2007). Preterm infants are at high risk of acquiring infections, and this is a significant contributor to mortality in this group. In Australia 3.3% of neonatal deaths are due to infection, but more than 70% of these infants are born preterm (Laws et al., 2007).

The disproportionate representation of preterm infants among infection-related neonatal deaths may be due to their immature immune systems (Wynn et al., 2009). Preterm infants have deficiencies in both innate and adaptive immunity, and in the interaction between these two systems (Durandy, 2003; Strunk et al., 2011). Preterm infants' immune systems may be further compromised, however, by factors associated with preterm birth. A variety of common pre- and postnatal events associated with preterm birth have the potential to modulate immunity.

## Preterm immune immaturity

**Figure 1:**
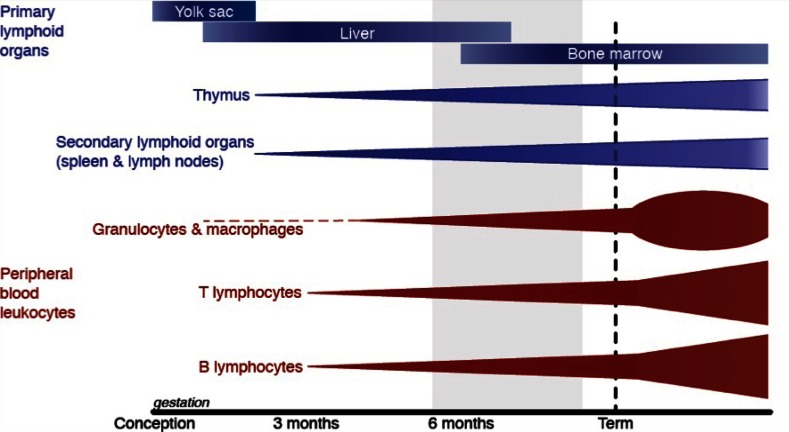
**Leukocyte development begins in the yolk sac before moving to the liver and finally the bone marrow.** The development and maturation of primary lymphoid organs (blue) and peripheral blood leukocytes (red) occur throughout gestation but is not complete until after birth. The light gray shading shows the gestational ages of preterm births from the threshold of viability (24 weeks; at which 50% of infants in developed countries survive) to 37 weeks of gestation. Based on a figure from Durandy (2003).

At the time of term birth, the immune system has not fully matured. The inexperienced adaptive immune system must still develop specificity and memory, which is completed only in the early childhood years (Hannet et al., 1992). As such, normal term neonates rely heavily on their innate immune response but this too is immature (Marodi, 2006). Immaturity of the immune system is more pronounced in infants born preterm (Figure 1).

### Innate immunity

The innate immune response of preterm infants is reduced in its capacity to adequately respond to infections due to deficiencies in the soluble protein/peptide and cellular responses to infection.

Soluble proteins and peptides have the ability to opsonize pathogens (to aid in phagocytosis) and to directly kill pathogens through their antimicrobial properties. There is limited production of soluble factors, such as immunoglobulin (Ig), by the fetus so it must rely on maternal supply. Antigen-specific IgG is transferred across the placenta from the maternal circulation in large amounts after 32 weeks of gestation (van den Berg et al., 2011). Transfer increases with fetal age; so preterm infants have low levels of circulating maternal IgG (Heininger et al., 2009; van den Berg et al., 2011). Low IgG levels result in a lack of opsonization, leading to deficiencies in phagocytosis (Källman et al., 1998).

Antimicrobial proteins and peptides (APPs) are released from leukocytes, including neutrophils, monocytes/macrophages and lymphocytes, and can bind to microorganisms and destroy them. APPs act through various mechanisms to destroy pathogens, such as iron binding (using the bacterium's source of iron), enzymatic destruction, zinc depravation and membrane pore formation (Levy, 2004). The production of APPs is positively correlated with gestational age (Strunk et al., 2011).

The classical, alternative and lectin complement pathways are all reduced in their pathogen-killing abilities in preterm infants (Fietta et al., 1987). Complement proteins act through various mechanisms to activate the C3 protein and induce phagocytosis. Preterm neonates are deficient in the production of C1, C4 (classical pathway) and factor B (alternative pathway) compared to term neonates (McGreal et al., 2012). Preterm infants are also deficient in the pattern-recognition receptor mannose-binding lectin (MBL), for which production increases as gestational age increases (Strunk et al., 2011; Sharma et al., 2012). MBL is produced by hepatocytes, and opsonizes pathogens, aiding activation of the complement system through the lectin pathway. Associated with MBL, L-ficolin production and function is also reduced in preterm neonates (Swierzko et al., 2009). These deficiencies in non-cellular responses to pathogens can lead to reduced phagocytosis and impaired pathogen clearance by phagocytes (Driscoll et al., 1990; Källman et al., 1998).

Phagocytes include neutrophils, monocytes/macrophages and dendritic cells (DCs). Preterm infants have a reduced pool of neutrophils and monocytes, and their precursors, due to reduced granulocyte colony-stimulating factor (G-CSF) and granulocyte-macrophage colony-stimulating factor (GM-CSF) levels (Carr and Modi, 1997). The relative monocytopenia and neutropenia of preterm compared to term neonates can greatly affect infants' ability to fight infection (Correa-Rocha et al., 2012), which can be improved by GM-CSF treatment although it does not reduce rates of sepsis in preterm neonates (Carr, 2000; Carr et al., 2009).

Neutrophils are among the first responders to infection and have an important role in bacterial clearance. In response to an invading pathogen neutrophils migrate to the sight of infection to digest and kill these microorganisms (Abbas and Lichtman, 2006). Neutrophils of preterm infants may have difficulty in migrating to sites of infection due to a reduction in the expression of adhesion molecules such as L- and E-selectin (Nussbaum and Sperandio, 2011). Neutrophils ingest, through phagocytosis, and kill pathogens by releasing enzymes from their cytoplasmic granules (Witko-Sarsat et al., 2000). Impairment in neutrophil function (phagocytosis, generation of oxygen radicals and intracellular killing of pathogens) is a risk factor for the development of sepsis (Carr, 2000). Extracellular pathogen killing is also limited in neonates, with reduced ability to produce neutrophil extracellular traps (NETs) (Yost et al., 2009). NETs are lattices made of DNA bound to granular and cytoplasmic protein, which are released from neutrophils to trap and kill pathogens (Brinkmann and Zychlinsky, 2012).

Monocytes are phagocytic blood-borne cells that differentiate into macrophages or DCs in tissue. Monocytes are capable of phagocytosis, have bactericidal mechanisms and are involved in antigen presentation to T cells (Abbas and Lichtman, 2006). Through the secretion of cytokines and chemokines and presentation of antigen, monocytes/macrophages can activate other immune cells such as B cells and T cells (Medzhitov and Janeway, 1997). The monocytes of preterm infants have reduced cytokine production, but a similar efficiency in phagocytosis and intracellular killing of pathogens as term neonates (Currie et al., 2011); however, they may be limited in their ability to activate the adaptive immune response because major histocompatibility complex (MHC) class II expression is reduced on leukocytes from preterm neonates (Perez et al., 2010), thus limiting their ability to present antigen to T cells and activate them.

### Adaptive immunity

Adaptive immunity, involving lymphocytes (B and T cells), is pathogen-specific and requires acquisition of immunological memory. Genetic recombination of lymphocyte cell surface receptors results in an increased repertoire of pathogen recognition. Recognition of a pathogen results in lymphocyte activation, pathogen clearance and memory lymphocyte production, leading to swifter adaptive immune responses on subsequent encounters with the same pathogen.

Maturation of adaptive immunity occurs mostly after term birth, so all newborn infants have deficiencies in T cell activation and cytokine production, B cell immunoglobulin production, and interactions between T and B cells, relative to adults.

Cell-mediated immunity involves two main types of T cells, cytotoxic T lymphocytes (CTL; CD8+) and T helper lymphocytes (Th; CD4+). T cells recognize pathogens when their T cell receptor (TCR) is presented peptide segments of a pathogen on MHC by an antigen-presenting cell (APC; macrophages and DCs) (Bujdoso et al., 1989).

CD8+ CTLs are involved in the eradication of intracellular pathogens such as viruses and are presented antigen by APCs expressing MHC class I. CD4+ T helper cells, activated by MHC II, are further divided into Th1 and Th2 CD4+ cells, defined by their cytokine profile. The Th1 phenotype is often classed as inflammatory, producing the cytokines interferon-γ (IFN-γ), interleukin (IL)-2 and tumor necrosis factor (TNF). The Th2 phenotype is anti-inflammatory, producing cytokines such as IL-4, IL-5, IL-13, and IL-10.

At birth lymphocyte subpopulations are lower than in adults, and require clonal expansion. This occurs in the first few weeks of postnatal life, with both term and preterm infants following the same pattern of expansion; but preterm infants have lower absolute numbers of circulating lymphocytes (Walker et al., 2011). A reduction in lymphocyte subpopulations is evident in children born preterm at 7 months (Berrington et al., 2005) and 8 years (Pelkonen et al., 1999) of age, when compared to age-matched children born at term. Preterm and term neonates have deficient T cell function as a result of a greater proportion of naïve T cells in the circulation and a low subpopulation of memory T cells (Walker et al., 2011). The increased proportion of naive T cells is a result of inefficient DC antigen uptake and presentation, and is contributed to by a reduction in MHC II expression on antigen-presenting cells (Strunk et al., 2011).

During fetal life, cytokine responses are driven toward a Th2 phenotype. The Th2 bias is thought to be a preventative measure against fetal rejection by the maternal immune system, with increased Th1 cytokine production linked to an increased risk of spontaneous abortion (Szekeres-Bartho, 2002). Preterm and term neonates are believed to be vulnerable to infection due to this bias toward a Th2 CD4+ T cell phenotype and as a result have lower production of cytokines such as IFN-γ in comparison to adults, which can result in deficient viral detection and clearance (Hartel et al., 2005; Marodi, 2006).

Compared to term infants, monocytes from preterm neonates have reduced production of TNF-α (Perez et al., 2010) and IL-12 (Tatad et al., 2008) following stimulation in culture. Monocyte production of IL-6 and TNF-α after cell stimulation is reduced in the fetus and neonate compared to adults (Holt and Jones, 2000; Strunk et al., 2004). Preterm monocytes have lower IL-6 and TNF-α production when the cells are unstimulated but after bacterial challenge production is comparable to monocytes from term infants (Tatad et al., 2008). Conversely, IL-8 production and baseline Toll-like receptor (TLR)-4 expression is greater for monocytes from neonates compared to adults. IL-8 production is lower in monocytes from preterm neonates compared to term neonates but monocyte TLR-4 expression is comparable (Levy et al., 2009). Monocyte production of IFN-γ and TNF-α in response to stimulation increases in an age-dependent manner and is lowest in preterm infants (Hartel et al., 2005).

Humoral immunity involves B cells, which recognize whole pathogen molecules through membrane-bound antibodies including IgM, IgG, IgA, and IgE. Immunoglobulins IgM and IgD are co-expressed on naive B cells; upon activation the B cells class-switch to express another antibody isotype and lose IgD expression. B cells, when activated, secrete antibodies to fight the pathogen by opsonization (Liu and Banchereau, 1997). In neonates the ability to class-switch is reduced, resulting in B cells secreting mainly IgM antibodies. B cell class switching is aided by T cell-dependent B cell activation, through CD40 and CD40L (CD40 ligand) binding. Neonatal T cells have reduced expression of CD40L, even when activated, resulting in a reduced production of the antibodies IgG and IgA by neonatal compared to adult B cells (Nonoyama et al., 1995). There is even greater reduction in the expression of CD40 and CD40L in preterm infants (Kaur et al., 2007).

## Perinatal infection and inflammation

Intrauterine inflammation is the principal cause of preterm birth (Hagberg et al., 2002; Romero et al., 2007; Fahey, 2008). Intrauterine inflammation can be caused by bacterial infection, which can occur by ascent of bacteria from the birth canal, crossing of the placenta or membranes by microbes, or inadvertent pathogen transfer into the amniotic cavity during amniocentesis (Hagberg et al., 2002; Goldenberg and Culhane, 2003). Intrauterine inflammation is inversely correlated with gestational age; it is present for 83% of infants weighing less than 1000 g at birth and for 10% in infants greater than 2500 g (Goldenberg and Culhane, 2003). Intrauterine inflammation affects as many as 30% of all neonates born at ≤34 weeks gestation (Lahra and Jeffery, 2004).

Intrauterine inflammation can increase the risk of infant mortality (Hagberg et al., 2002; Fahey, 2008). Clinical and experimental studies demonstrate that bacteria or pro-inflammatory mediators in amniotic fluid can be inspired or swallowed by the fetus to elicit a fetal inflammatory response (FIRS) (Gotsch et al., 2007), which is also observed in a sheep model of intrauterine inflammation (Moss et al., 2002c). In sheep, intrauterine inflammation increases the risk of perinatal neurological injury (Duncan et al., 2006; Nitsos et al., 2006) and impaired gut development (Wolfs et al., 2009). The fetal sheep lung has increased surfactant production and improved compliance after exposure to inflammation (Moss et al., 2002a), consistent with reduced rates of respiratory distress syndrome (RDS) in preterm human neonates exposed to inflammation *in utero* (Lahra and Jeffery, 2004). However, at least in the sheep, lung structure is simplified (Moss et al., 2002b). Simplified lung structure arising from prenatal exposure to inflammation could contribute to a potential increase in the risk of bronchopulmonary dysplasia (BPD) in human neonates (Been et al., 2009).

FIRS is characterized by an increase in fetal plasma IL-6, C-reactive protein, IL-1, IL-8, and GM-CSF (Berry et al., 1995; Goldenberg et al., 2000; Gotsch et al., 2007). There is evidence of monocyte and neutrophil activation in addition to increased numbers of these cells in the fetal circulation after exposure to inflammation in humans and sheep (Kallapur et al., 2007; Kramer et al., 2007; Romero et al., 2007). Lymphocytes are activated during infections *in utero* in humans, indicating the fetal adaptive immune response is at least partly responsive (Duggan et al., 2001). Fetuses and neonates exposed to intrauterine inflammation have increased Th1 cells corresponding with an increase in IFN-γ, indicating a potential shift from Th2 to Th1 of the fetus. The shift to Th1 cytokines may lead to membrane rupture because normal term labor is partially an inflammatory event, with an increase in the production of Th1 cytokines TNF-α, IFN-γ, IL-1β, and prostaglandins in the fetal membranes and amniotic fluid (Sykes et al., 2012). Intrauterine inflammation also increases production of these cytokines (Romero et al., 2011) and prostaglandins (Westover et al., 2012).

Animal experimentation has been valuable for understanding the immune consequences of intrauterine inflammation. Immune cells (including monocytes, neutrophils, and lymphocytes) in fetal sheep lung tissue significantly increase in response to intra-amniotic lipopolysaccharide (LPS) infusion (Kallapur et al., 2007; Kramer et al., 2007). Fetal thymic cell populations are altered after LPS exposure, resulting in a decrease in CD8 and MHC II expression on thymocytes (Melville et al., 2012); however, LPS up-regulates MHC II expression on circulating fetal monocytes (Kramer et al., 2005). The effect of LPS on MHC II appears to be tissue-dependent. In the thymus MHC II is not functioning to activate T cells, but is involved in selection and development of T cells. Thus, the reduction in MHC II may lead to altered CD4 production. Peripherally, activation of leukocytes resulting in MHC II up-regulation would aid in eliminating the threat. The increased number of lymphocytes and expression of MHC II may indicate that the fetus is capable of reacting to infection with an adaptive immune response, along with these innate responses, consistent with observations in humans (Duggan et al., 2001). Functional “maturation” of the immune system may be a consequence of intrauterine inflammation because preterm monocyte hydrogen peroxide and cytokine production increases after infusion of LPS into the amniotic cavity in sheep (Kramer et al., 2005, 2007; Kallapur et al., 2007). Responsiveness of the preterm immune system to intrauterine inflammation is demonstrated further by the observation that repeated pro-inflammatory exposures induce tolerance in preterm sheep. Repeated doses of LPS into the amniotic cavity of sheep, at 2 and 7 days before preterm delivery, cause a reduction in IL-6 secretion in fetal sheep when compared to a single dose of LPS (Kallapur et al., 2007). This tolerance effect clearly demonstrates modulation of the immune system in response to the initial stimulus.

Deficiencies in preterm immune function have implications for the eradication of postnatal infections. Particularly important are nosocomial infections, which occur more frequently in preterm infants due to their extended hospital stays. Mortality associated with early-onset sepsis is increased in preterm infants, and rates correlate inversely with gestational age. Of the infants born after exposure to chorioamnionitis, 4.8% develop sepsis (Soraisham et al., 2009) and 16% of infants with sepsis die (Adams-Chapman, 2012). Intrauterine inflammation increases the risk of early-onset sepsis, likely because at least some of these postnatal infections originated *in utero*. However, intrauterine inflammation decreases the risk of late-onset sepsis (Strunk et al., 2012), potentially because these infants experience immune “maturation” by the earlier (intrauterine) exposure to infection, or because they require less respiratory support and accompanying invasive care.

## Preterm birth and antenatal glucocorticoids

Antenatal glucocorticoids have been utilized clinically for prevention of neonatal respiratory disease in preterm infants for ~30 years. In 1969, Liggins used the synthetic glucocorticoid, dexamethasone, to induce preterm labor in pregnant ewes and found that the lambs born after dexamethasone were able to breathe spontaneously, whereas preterm lambs that had not received dexamethasone could not (Liggins, 1969). Since this time, numerous randomized controlled trials have demonstrated beneficial effects on the preterm respiratory, central nervous, and gastrointestinal systems of antenatal glucocorticoids (Roberts and Dalziel, 2006).

Glucocorticoids are used in adults to modify immune responses in allergy and autoimmune diseases but the equivalent effects on the fetal immune system are not well-understood (Tuckermann et al., 2005).

The hypothalamic-pituitary-adrenal (HPA) axis regulates many physiological processes, including the immune system. An increase in pro-inflammatory cytokines causes activation of the HPA axis, which increases cortisol. Cortisol suppresses the immune system, by acting on glucocorticoid receptors of leukocytes, which causes translocation to the nucleus (Beato et al., 1995) and interference with transcription of pro-inflammatory factors (NF-κB and STAT) (Tuckermann et al., 2005). Interference with these transcription factors reduces cytokine production and prevents the immune system overwhelming the body with inflammation (Barnes, 2005). Glucocorticoids can also induce apoptosis of DCs and lymphocytes (Barnes, 2005).

The effect of an increase in endogenous glucocorticoids (via stress) or maternal administration of exogenous glucocorticoids on the developing immune system has not been extensively studied in humans or other animals but the limited data show lasting effects. Glucocorticoid exposure results in long-lasting alterations to physiological and cellular responses to infection and inflammation in the offspring. In pigs, maternal cortisol treatment during pregnancy caused an increased febrile response of female offspring to LPS challenge (de Groot et al., 2007). Administration of adrenocorticotropic hormone to pregnant Rhesus monkeys over a 2-week period alters immune responses of the juvenile offspring, reducing their febrile and cytokine responses to IL-1 (Reyes and Coe, 1997). Induction of maternal stress (causing an increase in maternal corticosterone) in rats resulted in a reduction in proliferation of certain subgroups of lymphocytes after mitogenic stimulation, in offspring at 8–9 weeks postnatal age (Kay et al., 1998).

Human clinical studies have observed a reduction in lymphocyte number in preterm neonates after antenatal glucocorticoids (Chabra et al., 1998; Kavelaars et al., 1999) but there appears to be an overall increase in total leukocyte count, specifically an increase in neutrophils (Fuenfer et al., 1987; Barak et al., 1992). Meta-analysis shows that antenatal glucocorticoids may reduce the risk of neonatal sepsis (Dembinski et al., 2003; Roberts and Dalziel, 2006). However, an association between increased early-onset neonatal sepsis and multiple-course maternal betamethasone treatment has been observed in one study (Vermillion et al., 2000). A recent study showed that the ability of neonatal cord blood leukocytes to produce cytokines is not altered with antenatal glucocorticoid treatment (Kumar et al., 2011).

Intravenous administration of dexamethasone to fetal sheep, in a dose approximating that used antenatally in women at risk of preterm birth, results in an increase in total leukocytes between three and 12 h after administration due to an increase in neutrophils, but a reduction in lymphocytes (Edelstone et al., 1978). In contrast, maternal intravenous dexamethasone administration does not alter fetal circulating leukocyte counts but increases maternal neutrophils and decreases maternal lymphocytes (Edelstone et al., 1978). Similarly, pregnant women administered antenatal glucocorticoids have increased circulating neutrophils coupled with a decrease in lymphocytes (Wallace et al., 1998).

Betamethasone administration to pregnant rats on gestational days 19 and 20 (term 22 days) caused a reduction in lymphocyte proliferation and IL-2 production in late-gestation fetuses and for up to six days after birth (Murthy and Moya, 1994). Natural killer (NK) cells also had reduced cytotoxicity, particularly those from male offspring (Kay et al., 1998). Functional changes are also observed in monocytes of lambs born after maternal betamethasone injection. Phagocytosis of apoptotic neutrophils, but not bacteria, by preterm sheep monocytes was initially decreased after maternal betamethasone but was increased 3-fold seven days after (Kramer et al., 2004). These differences in phagocytic capacity may be explained by the observation that TLR-4 expression is not altered by glucocorticoids (Cepika et al., 2010), thereby not influencing detection of gram-negative bacteria. Overall, there appears to be an initial characteristic anti-inflammatory effect of glucocorticoids in the fetus, followed by “maturation” of immune function. Similar results are seen in adult monocytes exposed to glucocorticoids. Human monocytes are altered to an anti-inflammatory phenotype aiding in the resolution of infections (Tsianakas et al., 2012). In murine monocytes, glucocorticoids increase the expression of receptors responsible for the phagocytosis of apoptotic neutrophils (Nilsson et al., 2012).

From the available studies it appears likely that antenatal glucocorticoids have a modulating effect on preterm immune function. What is unknown is whether these effects are transient or persist into childhood and beyond.

## Mechanical ventilation

Preterm infants are born before completed development and maturation of the lungs. Therefore, they have a thick blood-gas barrier, undifferentiated or immature airway epithelium and reduced ability to produce surfactant (Pringle, 1986). They also may have difficulty clearing lung liquid, resulting in lungs with a reduced ability for gas-exchange and poor compliance, making them prone to collapse (Jobe et al., 2008). Reduced surfactant production, impaired gas exchange and inefficient independent respiration can result in the need for mechanical ventilation (Brown and DiBlasi, 2011).

Mechanical ventilation of neonatal lungs can cause ventilation-induced lung injury (VILI). In preterm infants, VILI is believed to be a major contributor to the development of the chronic respiratory disease BPD. The shear stress, inspiratory volume, air pressure, and oxygen concentration of ventilation are believed to cause epithelial cell damage, which contributes to protein leak into the airways, inhibiting the function of surfactant and increasing inflammatory cell infiltration (Attar and Donn, 2002; Hillman et al., 2007; Jobe et al., 2008). The most common inflammatory cell infiltrate is neutrophils, which migrate into the airways (Jobe et al., 2008; Hillman et al., 2009). Inflammation caused by stretch injury in preterm sheep results in elevated pulmonary mRNA expression of serum amyloid A3, IL-1β, and IL-6 with 1 h of ventilation (Hillman et al., 2011). Interleukin-6 and IL-8 increase as early as 20 min after ventilation (Wallace et al., 2009).

Ventilation of the preterm lungs also results in a systemic inflammatory response. Circulating phagocytes undergo activation in mechanically ventilated infants, resulting in increasing expression of the cell adhesion molecule CD11b on both neutrophils and monocytes (Turunen et al., 2006). Activation of CD4 and CD8 T cells occurs in preterm infants with RDS requiring mechanical ventilation, and there is increasing expression of CD54 and decreasing CD62L in the first three postnatal days (Turunen et al., 2009). Lymphocyte activation is further increased in preterm infants that develop BPD (Turunen et al., 2009). However, the absolute lymphocyte number is decreased in preterm neonates with BPD, contributed to by a decrease in circulating CD4 cells (Ballabh et al., 2003).

The changes in the activation of circulating leukocytes are coupled with increased cytokine production. Term infants that require mechanical ventilation have an increased serum plasma level of IL-8, IL-1β, and TNF-α, no change in IL-6, and a decrease in IL-10 (Bohrer et al., 2010). Extended duration on a ventilator increases the magnitude of pro-inflammatory cytokine proteins detected in serum from ventilated preterm neonates (Bose et al., 2013). Preterm infants that go on to develop BPD following mechanical ventilation also have increase serum levels of TNF-α and IL-6, but decreased IL-10 (Koksal et al., 2012), indicating that sustained systemic inflammation may be a risk factor for developing chronic lung diseases.

## Caesarean section and preterm birth

Rates of birth by Caesarean section have risen in developed countries in recent years, such that this mode of delivery now accounts for almost one-third of births (Li et al., 2011; Martin et al., 2011). The perinatal risks of Caesarean delivery for the mother and newborn are well-appreciated but there is emerging evidence of an impact of Caesarean section on long-term immune function.

A relationship between birth by Caesarean section and increased risk of asthma was first demonstrated just over a decade ago (Xu et al., 2000, 2001) and has since been confirmed by meta-analysis of over 20 individual studies (Thavagnanam et al., 2008). Some studies have shown an increased risk for atopy in children born by Caesarean section (Maitra et al., 2004; Renz-Polster et al., 2005; Salam et al., 2006; Pistiner et al., 2008; Kolokotroni et al., 2012), leaving them at an increased risk for hypersensitivity (Pistiner et al., 2008; Kolokotroni et al., 2012) but the relationship is not straightforward. Kolokotroni et al. found an increased risk of atopy in children born by caesarean section only for families with a history of allergic disease (Kolokotroni et al., 2012).

Relationships between Caesarean delivery and increased risk of asthma and allergic disease have been attributed to differences in microbial colonization of infants at birth (Gronlund et al., 1999) and the influence of gastrointestinal flora on development of the immune system (Siggers et al., 2011). Infants delivered by Caesarean section have lower diversity of gut microflora at 3 days of age than those delivered vaginally (Biasucci et al., 2010). Recent consideration of the human body not as an individual but as a symbiotic mix of human and microbial cells, with optimal function determined by the microbiome of the host, suggests that Caesarean section may contribute to a myriad of postnatal diseases, simply by influencing gastrointestinal colonization at birth.

The systemic immune function of neonates may also be impacted by Caesarean section because more than half of Australian women who deliver in this way do so without labor (Li et al., 2011). Newborn monocyte expression of TLR-2 and -4, critical mediators of innate immunity, is reduced in the absence of labor (Shen et al., 2009), potentially reducing the responsiveness of neonates to bacteria and viruses.

## Conclusion

Advances in obstetric and neonatal medicine have enabled profound reductions in perinatal mortality in recent years but this benefit has not come without cost. With increased rates of survival of preterm infants has come growing numbers of babies with illness and long-term disability, even for those born close to term (Cheong and Doyle, 2012). The consequences for immune development and function of preterm birth are largely unknown. We need to better understand the impact of preterm birth and associated factors, including obstetric and neonatal management, on immune function in order to improve health outcomes for the increasing number of individuals born preterm.

### Conflict of interest statement

The authors declare that the research was conducted in the absence of any commercial or financial relationships that could be construed as a potential conflict of interest.
